# Protocol: Chromatin immunoprecipitation (ChIP) methodology to investigate histone modifications in two model diatom species

**DOI:** 10.1186/1746-4811-8-48

**Published:** 2012-12-07

**Authors:** Xin Lin, Leïla Tirichine, Chris Bowler

**Affiliations:** 1 Ecole Normale Supérieure, Institut de Biologie de l’ENS, IBENS, Inserm, U1024, CNRS, UMR 8197, Génomique, Environnementale et Evolutive Section 3 CNRS UMR8197, 46 rue d’Ulm, Paris, 75005, France

**Keywords:** *Phaeodactylum tricornutum*, *Thalassiosira pseudonana*, Histone modifications, Chromatin immunoprecipitation, Epigenomics

## Abstract

In this report we describe a chromatin immunoprecipitation (ChIP) protocol for two fully sequenced model diatom species *Phaeodactylum tricornutum* and *Thalassiosira pseudonana*. This protocol allows the extraction of satisfactory amounts of chromatin and gives reproducible results. We coupled the ChIP assay with real time quantitative PCR. Our results reveal that the two major histone marks H3K4me2 and H3K9me2 exist in *P. tricornutum* and *T. pseudonana.* As in other eukaryotes, H3K4me2 marks active genes whereas H3K9me2 marks transcriptionally inactive transposable elements. Unexpectedly however, *T. pseudonana* housekeeping genes also show a relative enrichment of H3K9me2. We also discuss optimization of the procedure, including growth conditions, cross linking and sonication. Validation of the protocol provides a set of genes and transposable elements that can be used as controls for studies using ChIP in each diatom species. This protocol can be easily adapted to other diatoms and eukaryotic phytoplankton species for genetic and biochemical studies.

## Background

Diatoms are a group of eukaryotic phytoplankton with a wide distribution and a large diversity in marine and fresh water ecosystems. It is estimated that there are between 10,000 and 100,000 extant diatom species [1]. Diatoms play essential roles in global biogeochemical cycles because they are believed to be responsible for 20% of global carbon fixation and 40% of marine primary productivity [2]. Diatoms capture CO_2_ through photosynthesis and act as a critical buffer against global warming by sequestering organic carbon in the ocean interior. They can live under different conditions in all oceans from the poles to the tropics, and are often the first group of phytoplankton to benefit from sporadic nutrient upwelling events, indicating their intrinsic adaptability to changing environments.

Completed whole genome sequences from three species, the centric diatom *Thalassiosira pseudonana,* and the pennate diatoms *Phaeodactylum tricornutum* and *Fragilariopsis cylindrus* are now available [3,4], ( http://genome.jgi-psf.org/Fracy1/Fracy1.home.html). Additional pennate species, the toxic *Pseudo-nitzschia multiseries, Fistulifera sp. and Seminavis variabilis* are also being sequenced and will provide an additional source for comparative genomics. *T. pseudonana* is widely distributed in marine environments and is of significant ecological importance. *P. tricornutum* on the other hand is considered to be of little ecological relevance but is the model system for pennate diatoms because of a long history of physiological experiments and the availability of a wide range of tools for reverse genetics [5,6]. Furthermore, a digital gene expression database ( http://www.diatomics.biologie.ens.fr/EST3/index.php) is available for both species.

The whole genome sequences have revealed a wealth of information about diatom genes. It was shown for example that diatoms have acquired genes both from their endosymbiotic ancestors and by horizontal gene transfer from prokaryotes. But while DNA is the substrate for mutations upon which natural selection can act, DNA sequence in itself may not explain adequately their ability to adapt to changing environments, and more flexible mechanisms based on epigenetic processes could provide additional control. These changes include DNA methylation and histone tail post-translational modifications that alter chromatin structure. Study of diatom epigenomes can therefore provide a more in depth look at the regulatory mechanisms underlying their natural phenotypic adaptability to environmental changes.

Several tools for studying chromatin have been developed. Among them, chromatin immuno-precipitation (ChIP) has become a powerful tool to detect *in vivo* interactions between a DNA-associated protein and genomic DNA. ChIP combined with microarray or massively parallel sequencing is used to study gene regulatory networks active during development and/or in response to the environment. ChIP is also a valuable tool for mapping genome wide epigenetic modifications such as histone marks, and has been used to characterize several eukaryotic genomes [7,8]. However, no ChIP protocol has been reported for marine phytoplankton. *T. pseudonana* and *P. tricornutum* were therefore chosen to set up a ChIP protocol in diatoms using two histone marks known to characterize active and repressive chromatin states in other organisms.

The principle of a ChIP procedure includes: (1) Cross linking of DNA and protein with formaldehyde to covalently combine DNA and attached proteins *in vivo,* (2) Fragmentation of the fixed chromatin to an average size of 500 bp ranging from 200 to 1000 bp, (3) Chromatin extraction by a succession of extraction buffers, (4) Immunoprecipitation with specific antibodies, (5) Purification of immune complexes after immunoprecipitation and reverse crosslinking, and (6) Analysis of bound DNA by PCR which involves comparison of the intensity of PCR signals from the precipitated template with positive and negative controls. Standard PCR on immunoprecipitated DNA from a specific genomic region provides a direct assessment of protein association with that region, whereas quantitative PCR can assess not only whether a protein binds to that region, but also further compare the relative abundance at different genomic regions.

The protocol described in this work was optimized for each of the steps described above. It is an adaptation of ChIP protocols used for yeast and *Arabidopsis*[9,10]. It is therefore not new in its principles but takes into consideration features inherent to diatoms such as their siliceous frustule, the cell wall and the chloroplast.

## Materials and methods

Reagents:

Formaldehyde 36.5% (Fluka cat. No. 200018)

Glycine (Sigma cat. No. 2412)

Sucrose (Sigma cat. No. S-0389)

EDTA (Sigma cat. No. E-4884)

2-MERCAPTOETHANOL (Sigma cat. No. 125 k0165)

SDS (EUROMEDEX cat. No. EU660)

Triton X 100 (Amnersham bioscience)

Magnesium chloride (Sigma cat. No. 7786-30-3)

Sodium chloride (Sigma cat. No. 7647-14-5)

Sodium Acetate (Sigma cat. No. 127-09-3)

Lithium chloride (Sigma cat. No. 7447-41-8)

Sodium deoxycholate (Sigma cat. No. D-6750)

IGEPAL CA-630 (Sigma cat. No. 043 K0654)

NaHCO_3_ (Prolabo cat. No. 2 778.293)

Complete protease cocktail Inhibitor (Roche cat. No. 11873 580 001)

Proteinase K (Ambion cat. No. AM2546)

RNase A (Fermantas cat. No. EN0531)

Dynabeads protein G (Invitrogen cat. No. 100.04D)

Dynabeads protein A (Invitrogen cat. No. 100.02D)

Ethanol

Phenol (CAROLO ERBA Cat. no. 108-95-2)

Chloroforms (Prolab cat. No. 22 716.296)

Glycogen (Fermentas cat. No. R0561)

SYBER® Green master mix (Life Technologies)

Equipment

Sonicator (Bioruptor® UCD-200 from Diagenode)

Vortex

Rotating wheel

Incubator

Freezers (−80 and −20°C)

Falcon tubes (50 ml)

Eppendorf tubes (1.5 and 2 ml)

Siliconized eppendorf

Magnet Dynal

Solutions

**Extraction buffer I:** 0.4 M sucrose, 1 mini tablet Roche per 50 ml, 10 mM MgCl_2_, 5 mM 2-MERCAPTOETHANOL, 10 mM Tris–HCl pH 8

**Extraction buffer II:** 0.25 M sucrose, 10 mM Tris–HCl pH8, 10 mM MgCl2, 1% triton, 1 mini tablet Roche diluted in 1 ml (for 10 ml), 5 mM 2-MERCAPTOETHANOL

**Extraction buffer III:** 1.7 M sucrose, 1 minitablet diluted in 1 ml (for 10 ml), 0.15% Triton X-100, 2 mM MgCl2, 5 mM 2-MERCAPTOETHANOL, 10 mM Tris HCl pH8

**Nuclei lysis buffer:** 50 mM Tris HCl pH 8, 10 mM EDTA, 1 mini tablet of protease inhibitors diluted in 1 ml (for 10 ml), 1% SDS

**ChIP dilution buffer:** 1% triton, 1.2 mM EDTA, 167 mM NaCl, 16.7 mM Tris HCl pH8 CRITICAL keep at 4°C.

**Low salt wash buffer:** 150 mM NaCl, 0.1% SDS, 20 mM Tris–HCl pH8, 2 mM EDTA, 1% Triton X-100 CRITICAL keep at 4°C.

**High salt wash buffer:** 500 mM NaCl, 0.1% SDS, 1% Triton X-100, 20 mM Tris–HCl pH8, 2 mM EDTA CRITICAL keep at 4°C.

**LiCl wash buffer:** 0.25 M LiCL, 1% IGEPAL CA-630, 10 Mm Tris–HCl pH8, 1 mM EDTA, 1% sodium deoxycholate CRITICAL keep at 4°C.

**TE buffer:** 10 Mm Tris–HCl pH8, 1 Mm EDTA CRITICAL keep at 4°C.

**Elution buffer:** 1% SDS, 0.1 M NaHCO_3_ CRITICAL keep at 4°C.

CRITICAL: All the buffers should be prepared fresh and when required, the addition of the protease inhibitors, should be done directly before using the buffer.

### Protocol

#### Harvesting cells and cross-linking

1. Grow *P. tricornutum* culture in 400 ml artificial sea water under the standard conditions until cell density reaches around 1 million cells/ml.

2. Add 11.27 ml of 36.5% of formaldehyde to the culture to get final 1% concentration in the whole medium.

3. Stop fixation by adding 2 M glycine (final concentration is 0.125 M) for 5 min at room temperature.

4. Wash the cells with PBS solution twice by centrifugation at 4000 rpm for 5 min at 4°C.

Comment: Fixed pellet can be stored at −80°C for several months.

#### Chromatin extraction and sonication

5. Add approximately 5 ml of Extraction buffer I to 50 ml culture pellet.

6. Leave the falcon tubes on ice for 5 min.

7. Spin the solution at 4000 rpm for 20 min at 4°C.

8. Gently remove supernatant and resuspend the pellet in 1 ml of Extraction Buffer II.

9. Spin at 10,000 rpm for 10 min at 4°C.

10. Remove supernatant and resuspend pellet in 300 μl of Extraction Buffer III.

11. In a clean eppendorf, add 300 μl of Extraction Buffer III. Take the 300 μl solution (resuspended pellet) from last step and carefully layer it on top of the clean 300 μl of Extraction Buffer III.

12. Spin at 13,000 rpm for 1 hour at 4°C.

13. Remove the supernatant and resuspend the chromatin pellet in 300 μl (or 200 μl if small pellet) of Nuclei Lysis Buffer. Resuspend the pellet by pipetting up and down and vortexing (keep solution cold between vortexing).

14. Sonicate the chromatin solution for 9 cycles 30 seconds ON and 1 minute OFF for each cycle on full power. Keep 5 μl for DNA extraction to check sonication efficiency.

CRITICAL Supernatant is usually less than 300 μl following sonication due to liquid loss.

15. Check the sonicated chromatin after reverse cross-linking and recovering the DNA on 1% agarose gel. The DNA fragment should be around 200 bp-1000 bp.

Comment: Sonicated chromatin can be frozen at −80°C for 3 months or can be used directly for immunoprecipitation.

#### Immunoprecipitation and reverse crosslinking

16. Spin at full speed the chromatin solution for 5 minutes at 4 degrees to pellet debris. Remove supernatant to a new tube.

CRITICAL It is important to remove 20 μl for Total DNA Control for INPUT.

17. Measure out the remaining volume of sonicated chromatin and bring volume up to 3 ml with ChIP Dilution Buffer. The point here is to dilute the 1% SDS to 0.1% SDS with ChIP dilution buffer.

18. Split the chromatin solution into 3 tubes (1 ml each).

Tube 1. With DNA A beads labeled H3K4me^2^

Tube 2. With DNA A beads labeled H3K9me^2^

Tube 3. With DNA A beads labeled No Antibody (mock)

19. For each IP, mix 45 μl beads A and 45 μl DNA beads G in siliconized tubes. Wash DNA beads twice with ChIP Dilution buffer and resuspend beads in 90 μl Chip dilution buffer. Split the 90 μl resuspended beads into 30 and 60 μl volumes.

20. Add 5 μl of your antibody to the siliconized eppendorf tube that contains mixed 60 μl beads for Ig capturing. Add the diluted chromatin solutions into the tubes that contain 30 μl mixed beads for preclearing. Leave tubes with gentle rotation at 4°C for 2 hours.

Comment: Using precleared chromatin and siliconized eppendorf tubes can significantly decrease negative control (mock) noise background.

21. Wash the beads-Ig complexes once by 1 ml ChIP dilution buffer and resuspend in 60 μl ChIP dilution buffer. Transfer the precleared chromatin into the beads-Ig complexes. Leave tubes with gentle rotation at 4°C overnight.

22. Wash the DNA beads-Ig-antigen complexes 4 times using Low Salt Wash Buffer, High Salt Wash Buffer, LiCl Wash Buffer and TE Buffer in sequence. Use 1 ml of each buffer per wash and wash twice per sample. One wash is quick without agitation and the other one is at 4°C with gentle rotation for 5 min.

Comment: Put all the wash solutions on ice prior to immune complex collection.

23. Elute immune complexes by adding 250 μl of Elution Buffer to the washed beads. Vortex briefly for mixing and incubate at 65°C for 15 min (mix tubes during incubation). Put tubes on the magnet Dynal and carefully transfer the eluate to another eppendorf tube (not siliconized tube) and repeat elution once again. Combine them to obtain final 500 μl of elute.

25. Add 20 μl 5 M NaCl to the eluate and reverse crosslink at 65°C overnight.

Comment: Do not forget to get your total DNA out of the freezer and reverse cross-link with other samples (Add 500 μl of Elution buffer + 20 μl 5 M NaCl).

#### DNA recovery

26. Add 10 μl of 0.5 M EDTA, 20 μl Tris–HCl 1 M (pH 6.5), 2 μl of 10 mg/ml proteinase K, and 1 μl of 10 mg/ml RNase to the eluate and incubate for one hour at 45°C.

27. Recover DNA by phenol and chloroform (phenol; phenol/chloroform 1:1; chloroform) extraction (equal volume) and precipitate DNA with EtOH (2 vol 1/2) and 1/10 vol NaAc (3 M pH 5.3). Add 2 μl glycogen (20 mg/ml) to ethanol precipitation step. Incubate at - 80°C for 15 min or 2 hours at −20°C.

28. Centrifuge at 4°C at 13,000 rpm for 30 min and wash pellets with 400 μl 70% ethanol. Dry the pellet at room temperature.

29. Resuspend the pellet in 50 μl of distilled water or 30 μl to concentrate DNA.

#### Quantitative PCR

For both diatoms, specific primers were designed for two genes and a set of TEs (Table 1). The input DNA pulled out from ChIP was diluted 10 times before q-PCR. Quantitative PCR was performed using a Roche LightCycler® 480 machine on 1 μl of IP, input and mock DNA which were mixed to 5 μl LightCycler® DNA Master SYBR Green I 2X, 3 μl forward/reverse primers 1 μM, and 1 μL H2O. The PCR program was performed as follows: 10 min at 95°C; 45 cycles of 95°C for 15 seconds and 60°C for 1 min.

**Table 1:** Primer sequences used for QPCR analysis

**Primer name**	**Sequence**
Tp H4 Promoter fw	AGCCTGATGGAGAGAGTGGA
Tp H4 Promoter rev	TACATCCAGGACCTCCGTTC
Tp H4 body fw	TCGTAGAAAACGGTCCCATC
Tp H4 body rev	CCTCCACTTGGAAGAAGCAG
Tp Phyto promoter fw	CGATGTTGGTTGAGTGTTGG
Tp Phyto promoter rev	GCGATGTGCTCTTTTTGACA
Tp Phyto body fwd	TTTGGATTCCGTGAGAAAGG
Tp Phyto body rev	GTCTCGTGCACTCATCTCCA
Tp EU432485 fw	CCAGAGCTCGACAAACATGA
Tp EU432485 rev	TCGTTTTCCCTACGTGGAAC
Tp EU432490 fw	AGGAACTCGGAGACAAAGCA
Tp EU432490 rev	ATGTGCCCTCTTCAACAACC
Tp EU432492 fw	GCTCTGTCGTCGGAAAACTC
Tp EU432492 rev	AGGACAGCCTGCGTAGAAAA
Tp EU432500 fw	TGATGCAACAGGACGAAGAG
Tp EU432500 rev	GCATTGTTGGCCTTGTACCT
Pt H4 promoter fw	GTTGGTCGTCCATCGTTAGC
Pt H4 promoter rev	CCGTGGACGTTCTTGGTAGT
Pt H4 body fw	AATTACCAAGCCCGCTATCC
Pt H4 body rev	GTTTCTGTGAAACGGCAGGT
Pt PHY promoter fw	CTTGCCATGTCTTTGCAGTG
Pt PHY promoter rev	GTCAACACGCAATCAAGCAC
Pt PHY body1 fw	CAGCGACGGAAATGGACTAC
Pt PHY body1 rev	TTAGCAAGCAAGTGCGTCAG
Pt BKB4022 fw	CGAAGCTACTATGCCGGAAG
Pt BKB4022 rev	AAGGACACGAGAGTCGAGGA
PTC30 fw	CGGACTTCACCGAAGACAAT
PTC30 rev	GAATGGCTTTGGCATCATCT
PTC66 fw	AGCGATGGAACATTGGTTTATC
PTC66 rev	AACGTATCGTGAGCCTGACC
PTC25 fw	GCCTACCCCATGAAAACTGA
PTC25 rev	AGGCTCACTCTGCCACTGAT
SCF Fw	CAGCCTGAGGCGAAAGATAC
SCF rev	TAGTTCTGACATGCGCCAAG

### Data analysis

The Ct value (number of cycles required for the fluorescent signal to cross the threshold) is recorded in the experimental report after analysis by Roche LightCycler® 480 software. The Ct values of the duplicates should show minimal variability, indicating that samples were properly handled (ideally, it should be below 0.2). Ct values were used for performing the calculation which consists on evaluating the fold difference between experimental sample and normalized input.

ΔCt (normalized to the input samples) value for each sample. ΔCt [normalized ChIP] = (Ct [ChIP] - (Ct [Input] - Log2 (Input Dilution Factor).

Where Input Dilution Factor = (fraction of the input chromatin saved)^-1^ × Input dilution factor before q PCR. Here the fraction of Input chromatin saved is 20 μl and the fraction for each IP is 90 μl. The IP fraction is 4.5 times the input fraction. For QPCR runs Input was diluted 10 times which makes the final dilution factor of the Input fraction (Input Dilution Factor) = 4.5 × 10 = 45. Then the equation above is as follows: ΔCt [normalized ChIP] = (Ct [ChIP] - (Ct [Input] - Log2 (45). Finally, the percentage (Input %) value for each sample is calculated as follows: Input % = 100/2 ^ΔCt [normalized ChIP]^. The “Input %” value represents the enrichment of certain histone modification on specific region.

### Peptide competition assay and western blot

The antibody was pre-incubated with the peptide prior to use in immunoblotting assays. Different amounts (depends on different peptides and antibody) of peptides were added to a 10 ml BSA solution containing antibody and incubated under gentle agitation for 4 h at room temperature and an additional 1 hour at 4°C before the immunoblotting assays. Calibration curve were performed for each antibody to determin the amount of antibody to use (data not shown). The antibodies and peptides used in this work include H3K4me2 (Millipore Ref: 07–030), H3K9me2 (Millipore, Ref: 17–681), H3K4me1 peptide (Abcam Ref: ab1340), H3K4me2 peptide (Abcam Ref: ab7768), H3K4me3 peptide (Abcam Ref: ab1342), H3K9me1 peptide (Millipore, Ref: 12–569), H3K9me2 peptide (Millipore, Ref: 12–430), H3K9me3 peptide (Millipore, Ref: 12–568). The references of H3K9me2 antibodies that were discarded because of the lack of specificity are Millipore Ref: 17–648 and Millipore ref: 05–1249. Different concentrations of antibody and peptide concentration were compared. The nuclear enriched protein used for immunoblotting was extracted following the chromatin extraction protocol with minor modifications: the culture was not fixed by formaldehyde and sonication was not needed.

## Results and discussion

### Chromatin extraction and processing

ChIP is a very powerful technique for revealing association of specific DNA regions with proteins of interest. However, it is not a trivial technique, and highly specific antibodies against the protein of interest or a particular histone modification are required. Furthermore, false-negative signals may originate from inefficient antibody binding, and the beads used in ChIP can bind non-specific sites and cause background noise in negative controls. The starting materials for immunoprecipitation are also critical in terms of number of cells in a given volume, and sample dilution can effectively decrease background noise. To avoid saturation of antibody in ChIP assays, calibration curves should be built before precipitation with the antibody to determine the optimal amount of antibody to use.

**Figure 1:**
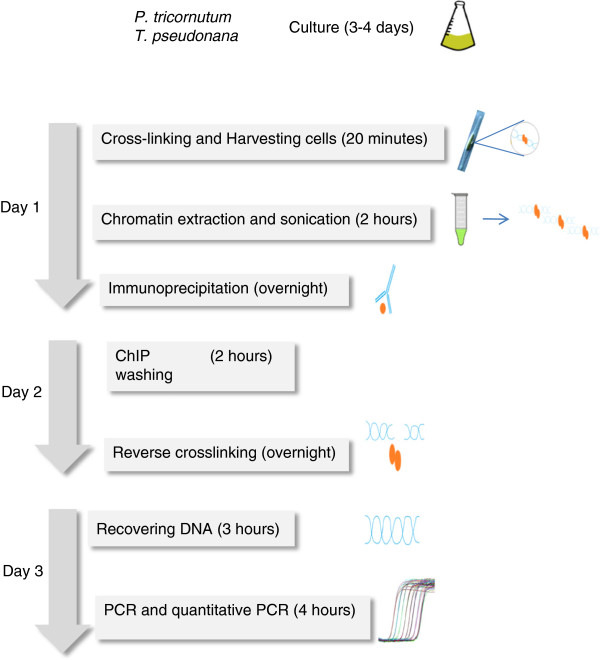
**Outline of the ChIP QPCR protocol.** Timing for each step is indicated between parentheses.

The protocol described herein (Figure 1) has taken into consideration these issues as well as the particularities of diatoms. Diatoms have the unique ability to precipitate soluble silicic acid into a finely patterned cell wall built from amorphous silica, and they also possess photosynthetic chloroplasts. Chromatin extraction buffers modified from *Arabidopsis* ChIP protocols were used for *P. tricornutum* and *T. pseudonana* which have plant features, such as chloroplasts and a cell wall. It is important in this protocol to use artificial sea water instead of natural sea water in order to control the different components of the medium such as silica. Extraction of chromatin from diatom species can be difficult because of the silica based rigid cell wall [11], which can interfere with chromatin extraction because it binds DNA. In this study, we have chosen species that have different requirements for silicon. *P. tricornutum* has a facultative requirement for silicic acid, whereas *T. pseudonana* needs a silicon source to grow. A growth medium without silicic acid was used for *P. tricornutum* while *T. pseudonana* grew in medium containing a relatively high concentration of silica (~ 88 μM) reflecting the amount found in natural environment.

To preserve cell integrity, diatom cells were fixed in the growth medium prior to any handling. Formaldehyde was used for cross linking. Cross linking is a time dependent procedure and our trials have established 10 minutes as an optimal time for both *P. tricornutum* and *T. pseudonana*. Excessive cross linking might reduce antigen accessibility and sonication efficiency. For a different diatom species, we recommend 5 minutes for a start, proceed with sonication, reverse cross link and run a gel to see how much DNA is recovered and whether the size is optimal. If not, we recommend trying longer or shorter times for cross linking.

**Figure 2:**
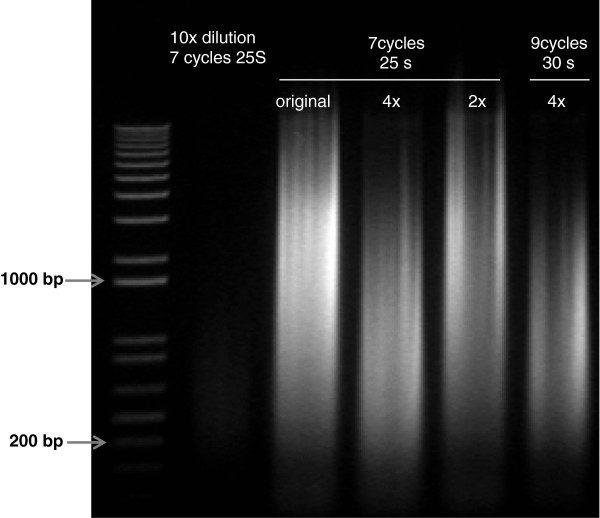
**Sonication efficiency.** Different times and number of cycles were used for sonication. After crosslinking, eluted DNA was loaded in a 2% gel to monitor the efficiency of sonication. From left to right, ladder, 10 times diluted sample containing 500000 cells/ml with 7 cycles 25 seconds ON and 1 minute OFF, original sample from 200 ml culture containing 5 million cells/ml, 4x and 2x diluted samples with 7 cycles, 25 seconds and 1 minute OFF and 4x diluted sample with 9 cycles, 30 seconds ON and 1 minute OFF.

Sonication time was determined after trying three different times, 7 cycles of 25 seconds ON and 1 minute OFF, 9 and 12 cycles with 30 seconds ON and 1 minute OFF. Nine cycles gave the best range of DNA sizes which is between 220 and 1000 bp with a maximum of DNA fragments at 500 bp (Figure 2). Different dilutions of 200 ml sample containing 5 million cells per ml were used for sonication as high cell density can interfere with the efficiency of sonication. Four times dilution gave the best range of fragment sizes (Figure 2).

### Peptide competition assay

**Figure 3:**
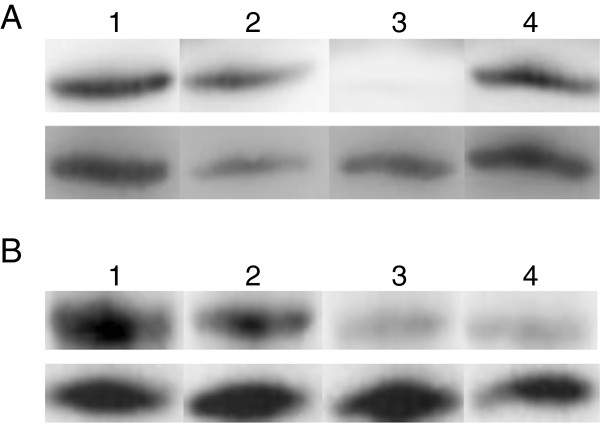
**Analysis of antibody specificity using peptide competition assays on western blots of *P. tricornutum *nuclear extracts.** (**A)** Upper lane contains from left to right 1 μg of H3K4me2 antibody alone or with 0.25 μg of one of the different modified peptides, H3K4me1, H3K4me2 and H3K4me3. Lower lane contains H4 antibody as internal loading control. (**B**) Upper lane contains 1 μg of H3K9me2 antibody alone or with 0.25 μg of one of the different modified peptides, H3K9me1, H3K9me2 and H3K9me3. Lower lane contains H4 antibody as internal loading control.

The nuclear-enriched protein used for immunoblotting was extracted following a chromatin extraction pro-tocol with minor modifications (see Methods section). Antibodies for two histone marks H3K4me2 and H3K9me2 were used for validation of the ChIP protocol. A peptide competition assay was performed to confirm the specific band reactivity of the antibodies. This is an important issue especially for genome wide studies as unspecific antibody binding will lead to non-specific signals increasing background noise and the occurrence of false positives. Both antibodies were pre-incubated with three different concentrations of the corresponding peptides (see Methods section) prior to immunobloting. For the H3K4me2 histone mark, the peptide competition assay did not detect the presence of a band for peptide H3K4me2 while a band was seen for the two other peptides, indicating the absence of competition with the other modifications of lysine 4 (Figure 3A). Three different H3K9me2 antibodies from Millipore were tested in this assay (see methods section for references). Two of them were discarded because of a lack of specificity while the third one gave better results in terms of specificity and noise (Figure 3B).

### H3K4me2 is enriched in genes in *P. tricornutum* and *T. pseudonana*

**Figure 4:**
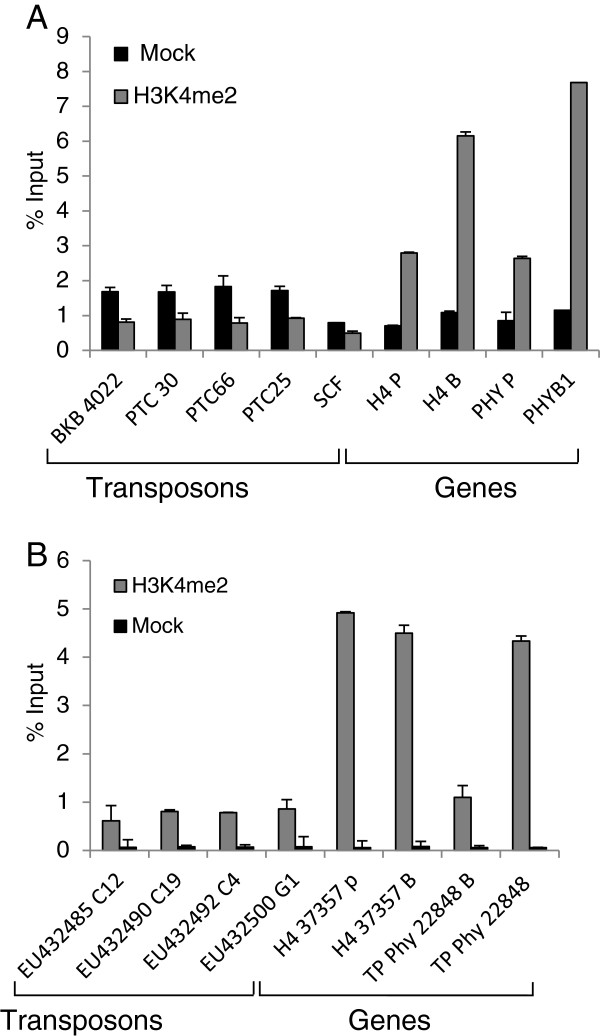
**H3K4me2 histone modification on different regions of genes and TEs in *P. tricornutum *(A) and *T. pseudonana *(B).** % IP indicates the enrichment. H4 P: promoter region of H4 histone gene. H4 B: body region of H4 histone gene. PHY P: promoter region of Dph gene. PHY B: body region of Dph.

Quantitative real time transcriptase polymerase chain reaction (Q-PCR) coupled with the ChIP protocol described herein was used to investigate the enrichment of H3K4 and H3K9 dimethylation on two genes, histone H4 and diatom phytochrome (Dph), and four transposable elements (TEs) in *P. tricornutum* and *T. pseudonana*. A clear enrichment of genes in H3K4me2 shown by a set of primers spanning promoter and gene body was demonstrated by Q-PCR (Figure 4A). Both histone H4 and Dph show significant differences in the enrichment in H3K4me2 between immunoprecipitated sample and mock which is the no antibody control. However, no significant differences were observed for the five TEs chosen in this study. Likewise, in *T. pseudonana*, the Dph and histone H4 show enrichment in H3K4me2 in both promoter and gene body, whereas the four chosen TEs (C12, C4, C19 and G1) show no enrichment in H3K4me2 (Figure 4B). Additional genes and TEs were tested and showed similar results (data not shown). Altogether, our data show a conservation of H3K4me2 location between the two diatom species and multicellular organisms, because genome wide studies in plants and mammals have indeed shown the presence of H3K4me2 on genes [12-14].

### H3K9me2 shows a different enrichment profile in *P. tricornutum* and *T. pseudonana*

**Figure 5:**
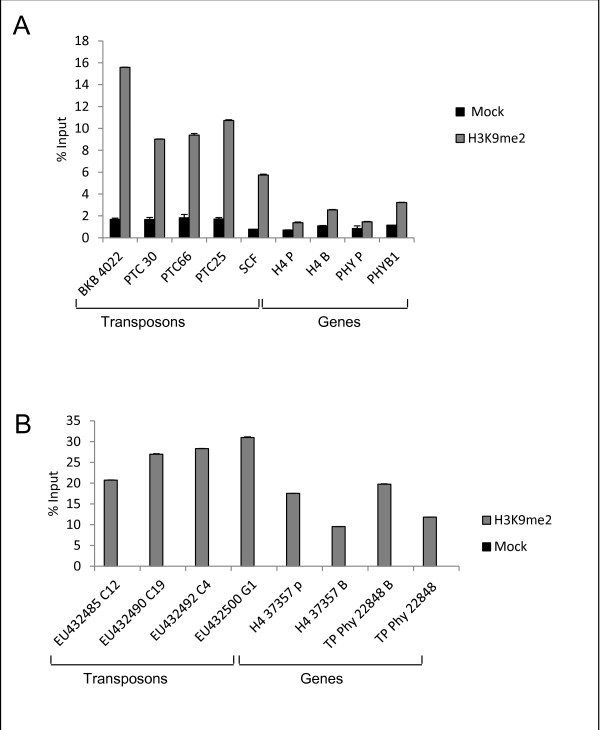
**H3K9me2 histone modification on different regions of genes and TEs in *P. tricornutum *(A) and *T. pseudonana *(B).** % IP indicates the enrichment. H4 P: promoter region of H4 histone gene. H4 B: body region of H4 histone gene. PHY P: promoter region of Dph gene. PHY B: body region of Dph. Mock values are indistinguishable from 0% in *T*. *pseudonana.*

ChIP analysis of H3K9me2 in *P. tricornutum* revealed that TEs contain a significant enrichment for this mark. We particularly focused on two previously characterized diatom-specific copia-like retrotransposable elements known as *Blackbeard* (*Bkb*) and *Surcouf* (*Scf*) [15]*Bkb* was particularly enriched in H3K9me2 while *Scf* was the least enriched. Intermediate levels were observed among the remaining TEs (Figure 5A). On the other hand, the two protein-coding genes histone H4 and Dph were clearly depleted of H3K9me2 in both promoter and gene body regions (Figure 5A). Both genes were shown by us and others to be transcriptionally active ( [5], unpublished results). It was previously shown that *Bkb* and *Scf* are also transcriptionally inactive in normal growth conditions [15]. This is consistent with the primary function of H3K9me2 in repressing TEs in order to maintain genome stability. In *P. tricornutum*, the ChIP assay shows that repressed TEs are marked by H3K9me2 while active genes are depleted in this mark which is similar to what has been observed in plants and mammals [16,17].

In *T. pseudonana*, a similar pattern of enrichment of TEs by H3K9me2 was observed (Figure 5B). The four tested TEs were highly enriched for H3K9me2, indicating a conserved profile for this mark among both diatoms. Surprisingly, some genes also showed a significant enrichment for H3K9me2, particularly at promoter regions (Figure 5B). This unusual association of H3K9me2 with active genes can be due to an intrinsic feature of the centric diatom *T. pseudonana* which is believed to have diverged from its distantly related pennate diatom *P. tricornutum* 90 million years ago. Comparative genomics and analysis of molecular divergence has shown indeed that both genomes are as different as those of mammals and fish [4]. Furthermore, combinatorial patterns of antagonistic chromatin marks are known to occur [18-21]. In *Drosophila* S2 cells, clusters of transcriptionally active genes were reported to be enriched in H3K9me2 [22]. Similarly, differentiated mouse ES cells were reported to contain large domains of H3K9me2 [23]. A hypothesis is that combinatorial chromatin marks can poise genes for transcription, creating more flexible chromatin states ready to adjust for subtle changes in the microenvironment of regulated genes [22,24]. The presence of chromatin marks known to be silent on euchromatin regions or active genes is intriguing and future analysis will be particularly important for elucidating this question.

## Conclusions

The ChIP protocol described herein provides reproducible results with two different diatom species grown in different media. The quality of the procedure monitored by the no antibody control is high as no or insignificant background noise was observed. This protocol is also rapid, and can be completed within 3 days. The quantity and quality of eluted DNA from immunoprecipitation are also satisfactory. Using the described ChIP method combined with real time quantitative PCR, we have demonstrated the existence in diatoms of two types of histone modifications, H3K4me2 and H3K9me2. The H3K4me2 mark is associated with transcriptionally active genes in *P. tricornutum* and *T. pseudonana*. This result is consistent with the distribution of H3K4me2 in plants and mammals. H3K9me2 binds TEs whereas no association with genes was detected in *P. tricornutum*. In *T. pseudonana,* H3K9me2 also correlated significantly with TEs, although it also appears to bind protein-coding genes. This is different from the distribution pattern of H3K9me2 in *P. tricornutum*. The differences of H3K9me2 distribution pattern between *P. tricornutum* and *T. pseudonana* may be inherent to the genetic and/or epigenetic background of the two species which belong to pennate and centric diatoms, respectively. Gene enrichment with H3K9me2 could be further confirmed by a genome wide study of H3K9me2 distribution. Our results show that our experimental and data analysis approach are indeed highly sensitive to detect differences between the two species if they do occur. The ChIP protocol we describe can be combined with microarray or massively parallel sequencing for further genome-wide studies. This protocol has been indeed successfully combined with Illumina sequencing for studies of global histone modifications in *P. tricornutum* (unpublished results). Furthermore, our ChIP assay can likely be easily adapted to other eukaryotic phytoplankton species for *in vivo* protein DNA interaction studies.
